# Annotations

**Published:** 1906-07-07

**Authors:** 


### ANNOTATIONS.

### Conscience and Society.

We have already in these columns drawn attention to the embarrassments which are besetting our laws with the gradual recognition of claims of conscience in the regulation of conduct. A case recently before an Old Bailey jury gives point to our observations. A member of the sect of Peculiar People was tried and sentenced to nine months' hard labour on the ground that he was guilty of manslaughter in the case of two of his children, by reason of his refusal to call in medical assistance during their illness. The judge, in his summing up, said that it would never do for each man to be a law unto himself, and that a religious conviction was no answer to such a charge. All persons were bound to take reasonable precautions to protect the lives of the children whom God had placed in their hands. Certainly this seems a sensible view of the duties of parentage, and the converse is susceptible of an easy *reductio ad absurdum*. But we fail entirely to see how such an attitude is to be reconciled with laws which permit the anti-vaccinationist to expose his children and all other unprotected people to the risks of small-pox, and the public to the expenses of their maintenance should they contract this disease. It is possible that the acumen of the legal profession may extract some subtle points of differentiation which will meet the circumstances, but to the plain man it seems that by the admission of conscience clauses in matters of public interest we have embarked upon an easy descent to Avernus which it will tax our law-givers to check. A rigid conscience is a hard task-master, both to its owner and to all who have to do with him. Well might Sir James Paget write laughingly of the students of whom he was warden: "I am glad to say that I do not think there is a strictly conscientious man in the College: they are all therefore nearly manageable."

### The Summer Diarrhœa of Infants.

The popular practice of grouping all cases of looseness of the bowels as "diarrhœa" has, of course, never satisfied the demands of medical science or practice, but it is only in recent years that there has been confidently recognised a variety of diarrhœa dependent on a specific infection and prevalent among infants during the summer months. The exact climatic conditions which determine the activity of the organisms on which this disease depends have been studied with considerable success, though they have not perhaps led to practical measures devoted to prophylaxis. The important point, so far as the practitioner is concerned, is that he may at any moment be confronted with a case which has very possibly been under domestic treatment for some time as a case of "simple diarrhœa," but which is essentially an example of an acute infective disorder. The frequent vomiting, the loose watery motions, and the general evidences of collapse are sufficient to attract the experienced eye and to secure recognition of a very serious disease. In some cases death occurs in the course of a few days, or even a few hours, after the onset. In others the process is less acute. But in all there is a degree of collapse not to be accounted for by a diagnosis of "simple diarrhœa" and calling for wise and energetic treatment. The routine prescription of astringent drugs is the reverse of wise. Indeed, in these cases, at the outset, castor oil and calomel are indicated. But the main demands are to meet the collapse for example, as by a hot bath, hot applications, transfusion of saline fluid, the hypodermic injection of strychnine, etc. Washing out the stomach and bowel may be practised, and milk is perhaps the most unsuitable of all foods. Attention to the necessary details, however, will readily follow when the possibility of an infantile diarrhœa being the expression of an acute infective disorder is borne in mind.

### Consumption Statistics.

Records of progress in the prevention and management of serious diseases are always welcome, both because the facts are gratifying in themselves and because also they lend encouragement and hopefulness to further efforts. An interesting illustration of this position is to be found in a recent official report made by Dr. W. S. Cook, medical officer of health for the borough of Greenock. From this it appears that during the last forty-five years the death-rate from consumption in Greenock has been reduced by 69 per cent., that is, from 44.6 per 10,000 inhabitants in 1860 to 12.0 and 13.9 in 1904 and 1905 respectively. The explanation of this gratifying state of matters is to be found less in measures directly attacking the disease than in the adoption of a policy calculated to promote the general health of the community. Amongst the features of this policy may be mentioned the adoption of an improvement scheme as a result of which a population of 3,217 was displaced from a crowded and unhealthy part of the town and was replaced by a population of 1,100 accommodated in new and sanitary dwellings. In addition, houses of less than 2,000 cubic feet capacity have been "ticketed" and these and common lodging-houses have been brought under regular inspection with a view to prevent overcrowding. The exact details, however, are not the points of main concern. The broad fact of importance is that improved sanitation, apart from specific attempts to destroy the bacillus of tubercle, have been sufficient to effect an enormous reduction in the death-rate from pulmonary phthisis. It is beyond question that this line of policy is far from being exhausted. Hence there follow two conclusions. First that by steady persistence in wise sanitation a still further control of tubercular disease may be gained; and secondly, that public money spent on sanitary measures produces not mere æsthetic and general advantages, but that its value is also to be found in a diminution of the incidence of specific diseases.

